# Extensive pyoderma gangrenosum-like lesions revealing a case of hyperzincemia and hypercalprotectinemia: when to suspect it?^☆^^☆☆^

**DOI:** 10.1016/j.abd.2018.12.001

**Published:** 2019-10-26

**Authors:** Ludimila Oliveira Resende, Marilia Formentini Scotton Jorge, Juliano Vilaverde Schmitt

**Affiliations:** a Faculdade de Medicina de Botucatu, Universidade Estadual Paulista, Botucatu, SP, Brazil; b Department of Dermatology, Faculdade de Medicina de Botucatu, Universidade Estadual Paulista, Botucatu, SP, Brazil

**Keywords:** Arthritis, Inflammation, Skin manifestations, Zinc

## Abstract

Hyperzincemia and hypercalprotectinemia is a rare inflammatory disease caused by a mutation in the PSTPIP1 gene, with a dysregulation of calprotectin metabolism. Calprotectin is a zinc-binding protein with antimicrobial properties and pro-inflammatory action. The authors report the case of a 20 year-old girl with cutaneous ulcers comparable with pyoderma gangrenosum, growth failure and chronic anemia, who was given the diagnosis of hyperzincemia and hypercalprotectinemia. Measurement of serum zinc and calprotectin concentrations are indicated in these cases.

## Introduction

Hyperzincemia and hypercalprotectinemia (Hz/Hc) is an autoinflammatory disorder characterized by chronic systemic inflammation, cutaneous lesions, arthralgia/arthritis, hepatosplenomegaly, pancytopenia, and growth failure, first described in 1985.^1^ The cause of this disease is related to a mutation in the proline-serine-threonine phosphatase-interacting protein 1 (PSTPIP1) gene, leading to dysregulation of the metabolism of calprotectin. This protein has zinc-binding capacity, as well as antimicrobial and proinflammatory activity. The literature presents few cases, and even fewer describe the dermatological aspects; furthermore, none of them are from Latin America (Table 1, Table 2).^2^, ^3^, ^4^

**Table 1:** Reports of Hz/Hc cases described in the literature with dermatological evaluation.^1^, ^3^, ^4^

	Previous reports								Current report
Age at diagnosis	18	18	9	14	35	21	10	5	20
Sex	M	M	F	M	F	M	F	M	F
Growth	Reduced	Reduced	Reduced	Reduced	Normal	Normal	Reduced	Reduced	Reduced
Hepatosplenomegaly	–	Yes	Yes	Yes	Yes	Yes	Yes	Yes	Yes
Skin lesions	PG	Vasc	No	No	Vasc, eczema	Vasc, ulcers	PG	Eyelid lesions	PG
Rheumatologic changes	–	Arthritis	Arthritis	Arthritis	Arthritis, uveitis	Arthritis	Arthritis	Arthritis	Arthritis
CRP (<10 mg/L)	–	41–143	100–200	22	17	45–146	60	108	35
Hemoglobin (g/L)	–	8	9	10.9	12.5	8	5.5	7.6	7.3
Zinc (10–18 μm/L)	120–197	180–200	82–96	160–200	175	77	183	152	138
Calprotectin (<0.001 g/L)	–	6.5	1.4–2.55	9	6.1	1.5	12.5	2.3	0.6

PG, pyoderma gangrenosum; Vasc, vasculitis; CRP, C-reactive protein.

**Table 2:** Summary of clinical characteristics of the cases of Hz/Hc with reported cutaneous lesions.

Feature	Value
*Age at diagnosis (years)*^a^	18.1 (8.7)
*Sex*	
Female	3/7 (43%)
Male	4/7 (57%)
*Hepatosplenomegaly*	6/6 (100%)
*Growth deficit*	5/7 (71%)
*Arthritis*	6/6 (100%)
*Anemia*	5/6 (83%)
*Hemoglobin (g/L)*^a^	8.2 (2.1)
*Serum zinc (μm/L)*^a^	153.4 (35.4)
*Calprotectin (g/L)*^b^	4.9 (1.5–6.1)

a Mean (standard deviation).

b Median (1st quartile − 3rd quartile).

## Case Report

**Figure 1:**
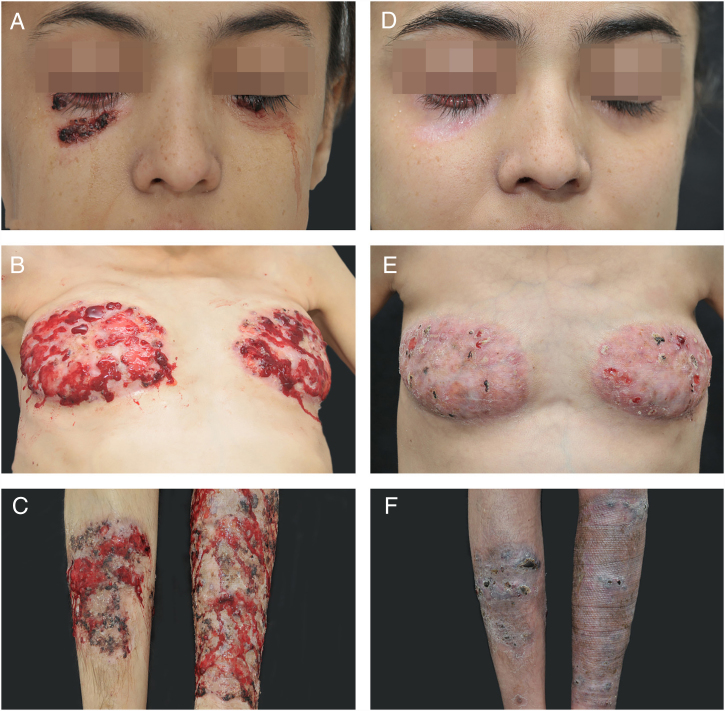
Skin lesions before (right) and after (left) six months of treatment.

A 20 year-old woman reported persistent ulcerated skin lesions with variable severity for five years, recurrent abdominal pain with episodes of diarrhea and joint pain in both knees accompanied by edema and local erythema since childhood. She reported chronic anemia that was unresponsive to several treatments. On the physical examination, the patient had short stature (percentile < 3%), cutaneous ulcerations with violaceous edges, interspersed by scar tissue and pustules with friable, bleeding and granular central area, together with the presence of purulent exudate and hematic crusts, affecting both lower eyelids, breast, and legs, in addition to a pronounced hepatosplenomegaly (Fig. 1A–C). The histopathological examination of the skin lesions was nonspecific, but compatible with pyoderma gangrenosum (PG). Initial investigations revealed pancytopenia, inversion of the albumin/globulin pattern (0.55; normal: 1.9–1.63), elevated C-reactive protein (CRP = 35; normal < 10 mg/L) and hepatosplenomegaly on ultrasonography, which also evidenced preserved parenchyma with extramedullary hematopoiesis in the liver biopsy histopathologic analysis. The patient also had portal hypertension with esophageal varices and pronounced osteoporosis, with a pathological T12.

Considering the diagnostic hypothesis of Hz/Hc, serum levels of zinc and calprotectin were measured: 869 μg/L (normal 70–120 μg/L) and 642 μg/L (normal < 1.6 μg/L), respectively.

Cyclosporin A (3.5 mg/kg/day) and prednisolone (1.5 mg/kg/day) were initiated, leading to wound healing, weight gain, and partial improvement of anemia, but the therapeutic response worsened after an attempt of waning, evolving with aggravation of preexisting lesions and development of an ulcerated lesion in the point of transcutaneous hepatic biopsy, compatible with pathergy (Fig. 1D–F). Immunosuppressive levels were restored, including a biweekly infusion of adalimumab 40 mg, with a significant but partial improve in the cutaneous lesions.

In the genetic assessment of the patient, the c.748G>A variant (Glu250Lys) was found in heterozygosis in the exon 1 of the PSTPIP1 gene. This variant is constituted by a substitution of glutamic acid for lysine at the codon 250 of the translated protein, which has been described as pathogenic in the Infervers and Clinvar databases (rs28939089) when associated with pyogenic arthritis, PG, and acne (PAPA) syndrome (MIM 604416).

## Discussion

Hz/Hc is a rare autoinflammatory condition, clinically characterized by pustular and ulcerative inflammatory cutaneous lesions, recurrent arthritis, hepatosplenomegaly, pancytopenia, and growth failure, with no predilection for gender, and usually diagnosed during youth. The disease is characterized by laboratory findings, such as extremely high serum levels of zinc and mostly calprotectin (500–12,000 times the normal levels).^3^

Calprotectin is formed by a complex of two MRP8/14 proteins that are endogenous activators of the toll-like receptor 4 (TLR-4) and belong to the family of the alarmins. This protein is found in the neutrophil cytosol and has intense pro-inflammatory properties.^5^, ^6^ Recently, a specific mutation resulting in a single amino acid substitution in the gene of the PSTPIP1 protein, a cytoskeleton-associated protein that modulates T-cell activation and IL-1b release, has been identified in these patients. Other mutations in this same gene had already been associated with other autoinflammatory diseases, such as PAPA syndrome, that has clinical similarities with Hz/Hc, but none of them present high levels of calprotectin and zinc.^2^

To date, no treatment has been fully effective, with reports of the use of corticosteroids, cyclosporine, tacrolimus, TNF, and IL-1 inhibitors. Hz/Hc is a chronic multisystem disease with nonspecific symptomatology and is present since childhood in most cases.^2^, ^4^, ^5^, ^6^, ^7^ Extremely high levels of serum zinc and calprotectin are strongly suggestive of the diagnosis; therefore, in suspected cases, serum level assessments should be considered.

The skin changes reported in Hz/Hc are vasculitis, eczemas, furuncle-like ulcers, and necrotic lesions. They tend to be inflammatory and ulcerated, sometimes pruritic, with clinical and histopathologic similarities to PG, usually affecting the lower limbs symmetrically, with reports of facial lesions, particularly on the eyelids. The observation of chronic lesions in young children and adults with hepatosplenomegaly, arthralgia/arthritis, high CRP, and microcytic anemia is suggestive of Hz/Hc.

## Financial support

None declared.

## Author's contribution

Ludimila Oliveira Resende: Elaboration and writing of the manuscript.

Marilia Formentini Scotton Jorge: Elaboration and writing of the manuscript; intellectual participation in propaedeutic and/or therapeutic conduct of the cases studied.

Juliano Vilaverde Schmitt: Effective participation in research orientation; intellectual participation in propaedeutic and/or therapeutic conduct of the cases studied.

## Conflicts of interest

None declared.

## References

[1] Hambidge K.M., Norris D.A., Githens J.H., Ambruso D., Catalanotto F.A. (1985). Hyperzincemia in a patient with pyoderma gangrenosum. J Pediatr.

[2] Holzinger D., Fassl S.K., de Jager W., Lohse P., Röhrig U.F., Gattorno M. (2015). Single amino acid switch defines clinically distinct proline-serine-threonine phosphatase-interacting protein 1 (PSTPIP1)-associated inflammatory diseases. J Allergy Clin Immunol.

[3] Sampson B., Fagerhol M.K., Sunderkötter C., Golden B.E., Richmond P., Klein N. (2002). Hyperzincaemia and hypercalprotectinaemia: a new disorder of zinc metabolism. Lancet.

[4] Isidor B., Poignant S., Corradini N., Fouassier M., Quartier P., Roth J. (2009). Hyperzincemia and hypercalprotectinemia: unsuccessful treatment with tacrolimus. Acta Paediatr.

[5] Ehrchen J.M., Sunderkötter C., Foell D., Vogl T., Roth J. (2009). The endogenous Toll-like receptor 4 agonist S100A8/S100A9 (calprotectin) as innate amplifier of infection, autoimmunity, and cancer. J Leukoc Biol.

[6] Stríz I., Trebichavský I. (2004). Calprotectin – a pleiotropic molecule in acute and chronic inflammation. Physiol Res.

[7] Sugiura T., Goto K., Ito K., Ban K., Okada S., Moriyama A. (2006). Effects of cyclosporine A in hyperzincaemia and hypercalprotectinaemia. Acta Paediatr.

